# Preparedness and Response Considerations for High-Consequence Infectious Disease

**DOI:** 10.3201/eid3108.250313

**Published:** 2025-08

**Authors:** Justin Chan, Corri B. Levine, Jocelyn J. Herstein, Nicole Cloutier, Lauren Sauer, Aneesh K. Mehta, Jared Evans, on behalf of the Basic and Translational State of the Science Working Group of the National Emerging Special Pathogens Training and Education Center’s Special Pathogens Research Network

**Affiliations:** New York University Grossman School of Medicine, New York, New York, USA (J. Chan); The University of Texas Medical Branch at Galveston, Galveston, Texas, USA (C.B. Levine, N. Cloutier); University of Nebraska Medical Center, Omaha, Nebraska, USA (J.J. Herstein, L. Sauer, J. Evans); Emory University School of Medicine, Atlanta, Georgia, USA (A.K. Mehta)

**Keywords:** high-consequence infectious disease, high-consequence pathogens, emerging pathogens, public health, emergency management, bioterrorism and preparedness, viruses

## Abstract

High-consequence infectious diseases (HCIDs) are acute human infectious diseases with high illness and case-fatality rates, few or no available effective treatment or prevention options, and the ability to spread in the community and within healthcare settings. Those characteristics lead to significant risks to patients and their close contacts, healthcare workers, laboratory personnel, and communities exposed to an outbreak. We describe aspects of healthcare system preparedness for and response to HCIDs, including the role of high-level isolation units, ensuring safe clinical laboratory capabilities and waste management, increasing availability of medical countermeasures, coordinating with stakeholders and systems of care, and communicating with the public. Finally, we discuss priority areas for further investment in HCID preparedness, care, and research. Effective and equitably disseminated medical countermeasures for HCIDs are urgently needed.

Pathogens that cause infectious diseases vary widely in virulence, infectiousness, and transmissibility. They are divided into different groups for ease of identification of threat to health and safety (Table) (*1**–**4*). The World Health Organization (WHO), the European Centre for Disease Prevention and Control (ECDC), the US Centers for Disease Control and Prevention (CDC), and US National Institutes of Health (NIH) play major roles in defining hazard classification and biosafety practices for a group of pathogens. NIH and WHO each defined 4 risk groups (RGs) for infectious agents and toxins on basis of the relative agent hazards involved when managed in a laboratory setting. Although slight differences exist between risk group definitions defined by NIH and WHO, RG1 (lowest risk) pertains to agents that are not associated with disease in healthy adult humans and RG4 (highest risk) contains agents that are likely to cause serious or lethal disease in persons and pose high community transmission risk (*4*). RGs are one consideration when conducting a biologic risk assessment to determine the Biosafety Level (BSL) in which the agent should be managed. CDC defines BSL-1–4 and describes laboratory design features, engineering controls, personal protective equipment (PPE), and biosafety practices that should be adhered to when handling a given biologic agent or toxin (*3*). BSL-4 practices and facility specifications apply to biologic agents that pose a high risk for life-threatening disease and for which there is often no available vaccine or therapy.

**Table:** Summary of major classification systems of infectious agents*

Term	Select agents and toxins	Category	Biosafety level	Risk group	Special pathogens and HCIDs (proposed)
Regulating or guiding organization(s)	CDC; USDA	US DOT Hazardous Materials Regulations	CDC; NIH	NIH	None
Levels of classification	SAT or Tier 1 SAT	A and B	1–4	1–4	NA
Scope of pathogens (animal vs. human vs. plant)	Pathogens and toxins posing a severe threat to animals, plants, and humans	Pathogens capable of infecting animals and humans	Pathogens capable of infecting animals and humans	Pathogens capable of infecting or causing harm to humans	Pathogens capable of infecting humans
Purpose of classification	Provide regulations on possession, use, and transfer of pathogens and toxins that have potential to pose a severe threat to the public, animal or plant health. The regulations allow laboratories to conduct important research on these materials in a safe and secure fashion.	Provide regulations on how to safely handle and transport infectious waste that may be capable of causing permanent disability or death in humans or animals upon exposure to the substance.	Provide guidance on the safe handling and containment of infectious microorganisms and hazardous biologic material to protect lab workers, the environment, and public from exposure to infectious microorganisms stored and handled in laboratories.	Describe relative hazard posed by infectious agents or toxins in the laboratory.	Provide guidance on human pathogens that require specialized clinical and public health response to prevent transmission in healthcare facilities and in the community.
Criteria related to classification	Pathogens and toxins that have the potential to pose a severe threat to public, animal or plant health, or to animal or plant products.	Category A classifies an infectious substance as in a form capable of causing permanent disability or life-threatening or fatal disease in otherwise healthy humans or animals when exposure to it occurs. Category B classifies an infectious substance as not in a form generally capable of causing permanent disability or life-threatening or fatal disease in otherwise healthy humans or animals when exposure to it occurs.	BSLs 1–4 are associated with specific guidance on how to prevent exposure to certain pathogens that could pose an infectious risk to persons working in a laboratory.	RG 1–4 are classified based on seriousness or lethality of human disease, whether preventive or therapeutic interventions are available, and risk posed to persons and the community.	Special pathogens cause HCIDs with high illness and death, have few available medical countermeasures, and can transmit from person to person.
List of pathogens	(*1*)	(*2*)	(*3*)	(*4*)	There is no universal list. Examples that meet criteria include Ebola, Marburg, and Nipah viruses.

*BSL, Biosafety Level; CDC, Centers for Disease Control and Prevention; DOT, Department of Transportation; HCID, high-consequence infectious disease; NA, not applicable; NIH, National Institutes of Health; RG, risk group; SAT, select agents and toxins; USDA, US Department of Agriculture; WHO, World Health Organization.

US federal agencies have designated specific infectious agents as requiring additional oversight of the possession and use of these pathogens. The Federal Select Agent Program (FSAP) is jointly managed by the Division of Regulatory Science and Compliance at CDC and the Division of Agricultural Select Agents and Toxins at the US Department of Agriculture (*1*). The program regulates the possession, use, and transfer of certain biologic agents and toxins, referred to as select agents, that could pose a severe threat to human, animal, or plant health. In addition, the US Department of Transportation (DOT) Pipeline and Hazardous Materials Safety Administration defines a classification system that outlines requirements for packaging and transporting certain infectious substances that can pose elevated risk to health, safety, and property during transport (*2*).

Those regulatory frameworks guide the safe handling of infectious pathogens in a laboratory setting, including waste management and transport. However, those classification systems do not specifically address risk posed when delivering care to patients in the clinical care setting. The National Emerging Special Pathogens Training and Education Center (NETEC) (*5*) describes a category of infectious agents and their associated diseases that pose elevated risk to staff, other patients, and the public and therefore generally merit management in a clinical setting with enhanced facility engineering controls and infection prevention and control processes. Several terms have been used to describe such pathogens, including highly infectious pathogen, highly hazardous pathogen, high-consequence pathogen, and special pathogen, among others. NETEC recommends using the term special pathogen to refer to the infectious agent (e.g., Ebola virus), and the term high-consequence infectious disease (HCID) to refer to the disease caused by that agent (e.g., Ebola disease). That terminology conveys the distinct nature of the infectious pathogens and concerns for the potential consequences to healthcare facilities, healthcare workers, and communities.

Clarifying terminology and describing characteristics of HCIDs can help focus appropriate resources to enhance clinical and public health responses during an outbreak and determine appropriate triage of individual patients to specialized facilities equipped for high-level isolation. Rather than providing a list of pathogens, we aim to describe defining characteristics of HCIDs, key aspects of HCID preparedness and response, and areas for advancement in HCID preparedness, care, and research. The list of specific pathogens that cause HCIDs will change over time because genetic evolution and medical advancements lead to changes in epidemiology, transmission, and clinical outcomes.

## Characteristics of HCIDs and Special Pathogens

NETEC agrees with other international health authorities (*6*–*8*) that HCIDs are acute human infectious diseases with high illness and case-fatality rates, few or no available effective treatment or prevention options, and the ability to spread in the community and within healthcare settings. As such, the use of a high-level isolation unit (HLIU), also known as a biocontainment unit (*9*), may be warranted to provide safe clinical care and prevent transmission to healthcare workers and other patients. Effective management of HCIDs typically requires enhanced coordination between stakeholders, including health systems and public health authorities, along with clear communication to maintain public assuredness about how the situation is being handled. A special pathogen is highly infectious, highly contagious, and highly hazardous and is likely to cause an HCID (*10*). Many special pathogens are on the WHO and NIH lists of priority diseases for research and development (*11*,*12*).

An example of a special pathogen is Ebola virus, which causes an HCID, Ebola virus disease. The disease has a high case-fatality rate of 32%–100% in past outbreaks (*13*) and significant person-to-person transmission. Although the US Food and Drug Administration has approved 2 monoclonal antibodies and 1 vaccine for the species *Orthoebolavirus zairense* (*14*), no medical countermeasures have been approved for the species *O. sudanense*, the second most common cause of Ebola outbreaks. Marburg virus (*15*) and Nipah virus (*16*) also demonstrate characteristics of special pathogens that cause HCIDs. Some emerging pathogens may be considered special pathogens, such as SARS-CoV-2 before effective medical countermeasures were available (*17*). Others can reemerge as special pathogens; monkeypox virus evolved to efficiently transmit between humans with limited availability of medical countermeasures and concern for a high case-fatality rate (*18*,*19*).

## Key Aspects of HCID Preparedness and Response

Outbreaks and cases of some HCIDs such as Ebola disease, Marburg virus disease, and Nipah virus infection have been more frequently detected and reported since the late 1990s (Figure). Those and other HCIDs demand purposeful preparedness and coordinated responses between frontline healthcare providers, health systems, public health agencies, the research community, and regulatory agencies. Together, the groups must develop a system of care that includes effective and efficient treatment facilities and protocols to safely manage infected patients, protect healthcare workers, and contain spread of the pathogen.

**Figure:**
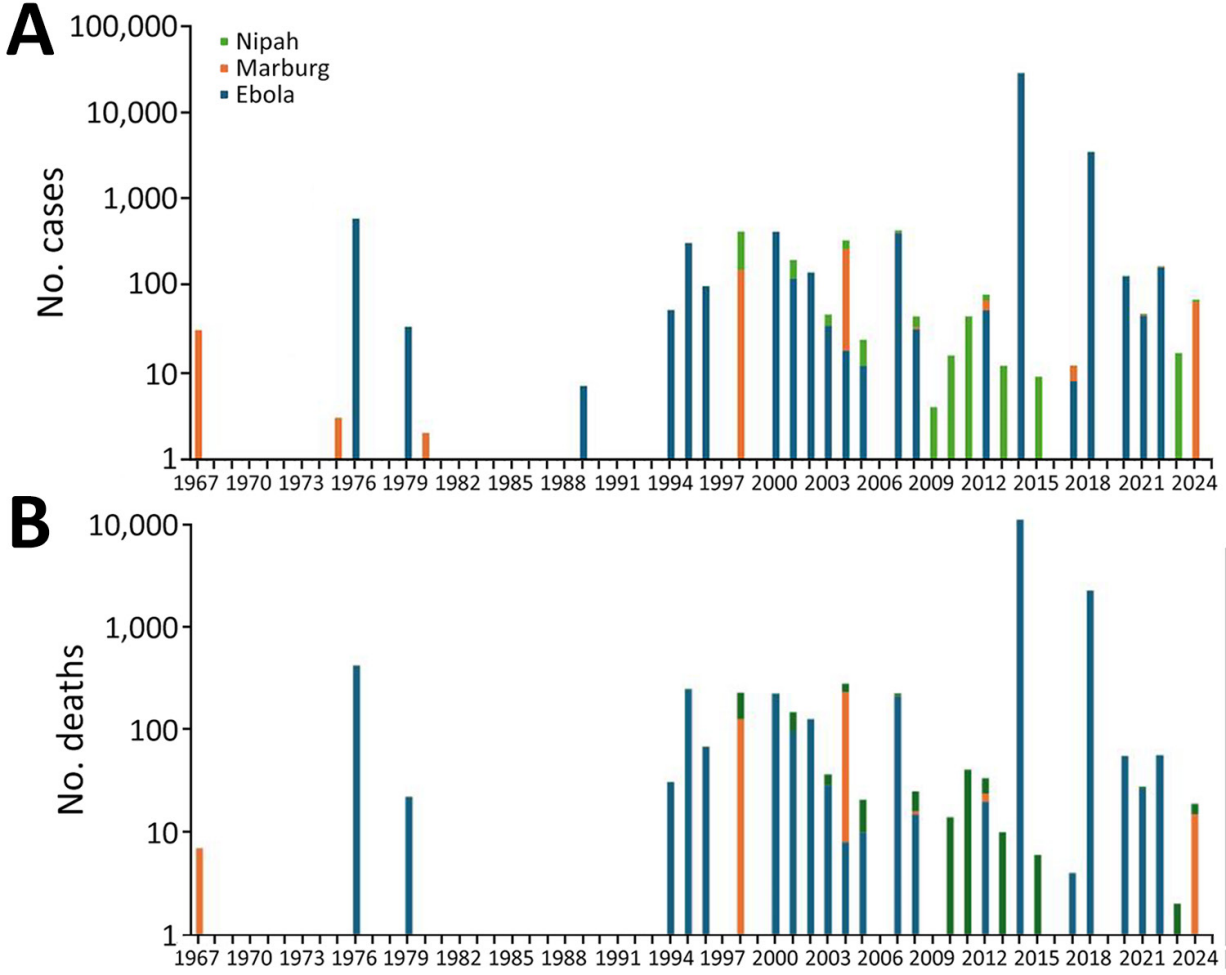
Global cases of infection with and deaths from Ebola, Marburg, and Nipah viruses, 1967–2024. A) No. cases; B) no. deaths.

### HLIUs

HLIUs constitute a key element of a response to HCIDs. Such specialized facilities offer advanced isolation and ongoing patient care to a small number of patients. They implement infection prevention and control protocols and standards that are beyond the usual capabilities of most hospital settings. Characteristics of HLIU design and function have been described previously (*8*,*9*,*20*,*21*). The units must maintain regular education and training on infection prevention practices, use of PPE, and clinical care protocols for patients with HCIDs. Units involve multidisciplinary teams including nurses, physicians, advanced practice providers, laboratory technicians, respiratory therapists, infection preventionists, industrial hygienists, and waste management technicians, among others.

A total of 13 federally designated Regional Emerging Special Pathogen Treatment Centers in the United States operate HLIUs, funded by the Administration for Strategic Preparedness and Response. A limited number of additional Special Pathogen Treatment Centers (SPTCs) operate HLIUs (*22*); patient care capacity can be overwhelmed by a surge in cases of a novel pathogen, as we saw early on during the COVID-19 pandemic. During outbreaks, health systems may need to rapidly increase capacity to safely care for patients with contagious pathogens (*23*) and modify patient care space to increase isolation capacity (*24*). Moreover, frontline healthcare facilities are the likely setting where patients with HCIDs will initially seek care. It is critical to prepare those sites to identify suspected cases, implement prompt isolation, and inform public health officials while providing stabilizing treatment before possible transfer to an HLIU (*25*). In the United States, as of July 1, 2024, the Joint Commission regulatory agency requires specific elements of frontline preparedness for HCIDs at all hospitals (*26*).

HLIUs capacity is limited because associated costs are high (*9*,*27*,*28*), but they provide significant value in return. HLIU patient rooms can be used for routine patient care as well as isolation and care during an HCID outbreak. In addition to maintaining training for HLIU staff, HLIU programs can deliver training to frontline staff at other hospitals to improve regional preparedness (*20*). HLIUs also advance research in areas such as infection prevention, human factors engineering, environmental engineering, and PPE, regardless of whether there is a current HCID outbreak.

### Clinical Laboratory Services

Laboratories associated with HLIUs require staff trained in safe handling and transport of specimens containing special pathogens (*29*,*30*); in addition, all frontline hospitals must maintain minimum routine laboratory capabilities critical for providing stabilizing care (*31*). Certain sample processing techniques may generate aerosols and risk staff exposure to a special pathogen if proper precautions are not implemented. Clinical laboratories should conduct risk assessments to identify potential hazards and mitigate risk through the use of engineering controls, such as primary containment equipment (e.g., biosafety cabinets, sealed centrifuge rotors), and appropriate PPE (*32*). If those safety measures are not in place in the existing core laboratories, then point-of-care testing may be an alternative option.

### Medical Countermeasures

The limited availability of medical countermeasures, including vaccines and therapeutics, is a key characteristic and challenge of HCIDs. For example, an expanded access investigational new drug protocol available to prescribe tecovirimat for nonvariola orthopoxvirus infections posed substantial regulatory and administrative burdens on patients and prescribers to access the drug during the 2022 multinational mpox outbreak (*33*). This experience highlighted the importance of fostering partnerships between academic medical centers and community hospitals to ensure prompt access to therapeutics that may be beneficial, but have not achieved full regulatory approval, while collecting appropriate data to determine the therapeutic efficacy and safety of such investigational therapies.

### Management of Contaminated Materials and Waste

Management of waste and contaminated materials associated with HCIDs often requires enhanced protocols to ensure safe disposal. Many special pathogens, including Ebola and Marburg viruses, are considered category A infectious substances by the US DOT (*2*) and have stringent requirements for safe packaging and transport. Management of associated waste requires detailed protocols and training of staff to adhere to DOT requirements and to ensure staff and patient safety (*34*). Other processes may involve handling material contaminated with a special pathogen, including daily cleaning and disinfection while caring for a patient, terminal cleaning and disinfection, management of spills, management of the deceased, and discharge of successfully treated patients. In general, waste containing special pathogens should be segregated at the point of generation and contained in leakproof, puncture-resistant containers that are clearly labeled as biohazardous. That waste must be decontaminated using methods such as autoclaving, incineration, or chemical disinfection before disposal. Personnel handling this waste should wear appropriate PPE and be trained in proper waste handling procedures and emergency protocols to manage accidental exposures or spills. Finally, adherence to relevant local, state, and federal regulations related to category A waste handling is essential. Although the proper handling and disposal of category A infectious substances remains a challenge in HCID response plans (*35*), The Joint Commission mandates that all hospitals implement protocols for proper waste management cleaning and disinfecting of patient care spaces, surfaces, and equipment contaminated with special pathogens (*26*).

### Other Clinical Considerations

Other aspects of HCID preparedness and response require consideration of infection prevention and control, ability to provide standard of care, occupational health, and health equity. Those processes include developing and implementing protocols for performing invasive procedures when necessary, monitoring healthcare workers after caring for an HCID patient or handling infectious material, safely transporting patients within and outside of the hospital setting, visitor management, and preparing postexposure plans for staff and community members, including postexposure prophylaxis and quarantine. Protocols for special populations such as children and pregnant persons often require additional planning, expertise, and attention to providing equitable access to care.

### Coordination with Other Stakeholders and the System of Care

Public health agencies on the federal, state, and local level play important roles in HCID response (*36*). They promote awareness of and surveillance for outbreaks of concern, coordinate patient triage to the most appropriate care setting, and develop guidelines and protocols for safe patient transport, contact tracing, and prevention of community transmission. The United States is engaging a national special pathogen system of care to improve national preparedness for HCIDs; it consists of a tiered system of healthcare facilities from frontline hospitals to regional treatment centers led by NETEC as coordinating body (*37*). NETEC has also identified gaps in HCID direct care delivery, communication and coordination, workforce capacity, training and education, research, data systems and technology, monitoring and evaluation, financial sustainability, and supply chain management (*38*). Improving equitable access to these aspects of HCID care is critical; NETEC’s analysis indicates that minoritized groups currently have less access to special pathogens treatment centers.

### Communication with the Public

Events involving HCIDs are likely to make news headlines, so it is important to proactively address the public’s concerns regarding local or international outbreaks. During an outbreak, the public should be informed about known risk factors for contracting the disease; the information can help mitigate transmission related to individual behavior and avoid stigmatizing vulnerable groups. When a patient is admitted to a facility with an HCID, the approach to communication is multifaceted regarding the information that should be shared and who should receive it. If a hospital incident command system is activated, it will likely be a unified command with a joint information center. A public information officer would be identified to coordinate regular updates to the public. Such updates may happen through the local public health official or the HLIU facility, or often both, with all communications coordinated through the command system. In addition, protected health information may need to be shared with response agencies and senior governmental leadership, which must be done cautiously, on a need-to-know basis, and only as permitted by the law.

## Areas for Advancement in Preparedness, Care, and Research

Sustaining research on HCIDs is critical to advance the health and safety of the workforce and community. Research efforts should focus on better understanding of pathogenesis, including molecular mechanisms of disease, development and testing of therapeutics and vaccines, and development of diagnostics that can be used rapidly in various settings. Not all clinical facilities have the capabilities to collect or process research specimens; partnering with other organizations such as an academic institution or academic medical center can support or enable this effort.

Limited availability or access to medical countermeasures is a major gap in HCID preparedness efforts. Vaccines and therapeutics are under development in both in vitro and in vivo pipelines, but transition to clinical trials is difficult because of sporadic outbreaks and limited number of affected persons, which can be perceived as a lack of need for these medical countermeasures. The COVID-19 pandemic demonstrated the ability to rapidly develop effective medical countermeasures when sufficient investment is made (*39*). A similar proportionate scale of investment has not been made for many high-priority HCIDs, including Crimean-Congo hemorrhagic fever, Ebola virus disease, Marburg virus disease, Lassa fever, and Nipah virus infection (*11*,*12*). Although we do not suggest that all HCIDs merit the same scale of investment as was mobilized for COVID-19, the pandemic demonstrated the value of sustained, proactive funding for preparedness infrastructure and medical countermeasures. The epidemic and potential pandemic risk for HCIDs, increased by global interconnectedness, climate change (*40*), and frequency of spillover events (*41*), indicates that strategic investments could provide a global benefit.

To evaluate countermeasures properly, it is necessary to prepare trials before an outbreak occurs. Phase I/II safety and immunogenicity trials should occur when possible, and plans and protocols should be drafted for efficacy testing that can be implemented early in an outbreak. During the 2013–2016 Ebola virus outbreak in West Africa, clinical trials were initiated late, and clinical efficacy could not be determined because enrollment did not meet goals (*42*,*43*). Although placebo-controlled trials may not be ethically appropriate, randomized trials of multiple alternative therapeutics can be beneficial, such as the PALM trial initiated during the 2018 Democratic Republic of the Congo Ebola disease outbreak (*44*). When a clinical trial is not possible, WHO’s monitored emergency use of unregistered and experimental interventions framework should be consulted to ensure it is ethically appropriate to administer specific therapeutics while data are collected to contribute to future decisions about efficacy (*45*). Facilities that are expected or preparing to care for HCID patients should include plans for safe and ethical participation in research trials and also understand the processes for obtaining investigational countermeasures (*33*).

Vaccines, and protocols for their study, should be prepared in advance to determine efficacy and potentially limit spread as new outbreaks occur. Ring vaccination refers to a strategy to vaccinate persons who have been in close contact with an infected person to prevent transmission. Trials that compare different ring vaccination arms can be effective tools, and the use of randomization to immediate or delayed vaccination groups mitigates concerns about inequitable access (*46*). As with other countermeasures, placebo-controlled vaccine trials are likely not ethical; possible treatment arms that can evaluate efficacy and implementation hurdles are randomization to receive different vaccines, vaccine combinations, or timing between doses. Longitudinal data collected from vaccine recipients can be used to monitor immunogenicity profiles over time and long-term effectiveness during subsequent outbreaks.

Developing and improving access to diagnostics must also be prioritized in countries both endemic and nonendemic for HCIDs. The ideal diagnostic test would be feasible to deploy at scale in low-resource settings where HCID outbreaks often start and would be able to detect a pathogen at the earliest sign of illness. It is appropriate to be judicious about testing in nonendemic countries to prevent escalations resulting from false positives; however, we must be able to enhance laboratory capacity during active outbreaks.

The continual emergence and reemergence of HCID threats require preparation for future events. Immediate concerns include highly pathogenic avian influenza, which always has pandemic potential; other proximate threats include novel pathogens with relatively low human-to-human transmission, such as Middle East respiratory syndrome coronavirus (MERS-CoV) and Nipah virus. However, introduction of those types of pathogens into a highly populated area could increase the potential for sustained human transmission (*47*). Prioritization for developing diagnostics and medical countermeasures can be guided by several factors: the pathogen’s potential for sustained human-to-human transmission, disease severity, evidence of geographic spread, potential for spillover, lack of existing medical countermeasures, and historical response delays. Expanded surveillance and new artificial intelligence tools to inform risk-assessment can support prioritization (*48*). Strategic investment in novel approaches to clinical research and study design, investment in platform technologies (e.g., viral vector vaccines, monoclonal antibody platforms), and maintaining pathogen-specific research readiness, including validated animal models, early-stage product candidates, and regulatory protocols, can permit rapid escalation of countermeasure development if an outbreak emerges. The goal is not to predict every threat but to build flexible, proactive systems that reduce the time between pathogen emergence and intervention deployment.

We believe that public sector funding, particularly from national governments and multilateral partners, remains essential given the limited commercial incentive to invest in rare but severe pathogens that cause HCIDs. Funding decisions should also account for global interconnectedness with strategic investments to develop capacity where these pathogens are endemic or at highest risk of emerging. Supporting early detection, research, and containment efforts in disease-endemic regions serves both humanitarian and strategic national interests. Considering the COVID-19 pandemic resulted in $16 trillion in economic loss in the United States alone by one estimate (*49*), any increase in investment in improving prediction analytics would provide value.

## Conclusions

The US federal government (Table) developed several classification systems to stratify risk of infectious agents in the context of laboratory research, specimen transport, and waste management. Through the lens of patient care delivery, we describe characteristics of a group of HCIDs that represent high threats to public health because of their high illness and case-fatality rates, limited availability of effective treatment or prevention options, and the ability to spread in the community and within healthcare settings.

Optimal preparedness and response require appropriate patient identification, isolation, treatment, and waste management, which might involve the use of HLIUs. Effective medical countermeasures must be developed and made accessible in an equitable fashion. In addition, clear communication and coordination across healthcare workers, health systems, and public health authorities is necessary to ensure public safety and assuredness. Priority areas for investment include research on new diagnostics and medical countermeasures, including clinical trials that should be planned before an outbreak. Because the evidence base to guide several aspects of preparedness and response we have described is limited, a modified Delphi method (*50*) could be used to establish consensus guidelines.
